# “Mild” COPD: What Spirometry Conceals!

**Published:** 2017

**Authors:** Denis E. O’Donnell

COPD is a common inflammatory disease of the airways, alveoli and microvasculature that is under-diagnosed in smokers at risk for the disease. The majority of COPD patients have mild airway obstruction. Symptomatic smokers with mild COPD are at higher risk for earlier mortality and poorer perceived quality of life than non-smokers. Moreover, dyspnoea and activity restriction are common among smokers with minor spirometric abnormalities. Tobacco-related inflammatory injury of the lungs manifests as heterogeneous physiological impairment with highly variable clinical expression. Thus, simple spirometry provides only a crude assessment of disease pathophysiology, especially in the early stages of the disease.

Several studies have shown consistent physiological abnormalities on oscillometry, together with abnormal configuration of the mid-volume maximal expiratory flow-volume loop and increased pulmonary gas trapping, which collectively point to the presence of extensive small airway dysfunction despite FEV_1_, plethysmographic lung volumes and resting inspiratory capacity being within the normal range.

Recent exercise studies in symptomatic smokers with or without mild COPD have highlighted that exercise limitation is common and is multifactorial but that respiratory factors such as increased dynamic mechanical constraints are contributory. Lung microvascular inflammation, disruption of the alveolar-capillary interface and reduced pulmonary perfusion are increasingly identified in smokers with minor spirometric abnormalities. In this context reduced ventilatory efficiency during exercise (high V_E_/VCO_2_ nadir) is common in mild COPD and mainly reflects high physiological dead space (rather than alveolar hyperventilation or reduced VT) and thus a preponderance of lung units with high ventilation-perfusion ratios. This presentation explores the clinical consequences of this heterogeneous physiological impairment in smokers with unremarkable spirometry.

